# The Story of the Insane from Year to Year

**Published:** 1894-07-07

**Authors:** 


### THE STORY OF THE INSANE FROM YEAR TO YEAR.

### BARNWOOD HOUSE ASYLUM, GLOUCESTER.

On January 1st, 1893, there were in residence 158 patients, and at the end of December the number was 156. The admissions were 33, the recoveries 16, and the deaths seven. The recoveries work out at 48·4 on the admissions, and the deaths at 4·4 on the average number resident. Compared with county asylums, the recovery rate is high and the death rate low; but then it is more than probable that a considerable amount of selection was used in the admissions. Drink is said to have been the cause of the insanity in two cases, and hereditary influence in 18. We are glad to see that nine of the admissions were taken in at "very low rates of payment"; but we see no table which gives the various sums paid by different classes of patients, nor even the lowest and highest sums paid, so we are left in the dark as to the meaning of the above words. The Commissioners refer to the "case of a lady, the subject of an order under the 116 section of the Lunacy Act, 1890; we heard with surprise that it was made upon affidavits only, and that she was not seen by a master in lunacy, and that she had but one week's notice of the proceeding of the order, during which time she endeavoured to obtain legal assistance, but through reference to a wrong quarter did not get it." There is no expression of doubt as to the insanity of the patient, and no blame could be attributed to Dr. Soutar for admitting the lady. We have ere now expressed strong adverse views as to the New Lunacy Act from a medical standpoint, and judging from the above it is anything but faultless from a legal point. When are we to get a good Lunacy Act? The committee apparently intend to turn out demented cases to make room for curable ones, and there is something to be said in favour of the step, although the first duty of the governors would seem to be the relief of patients of slender means who have seen better days and who belong to Gloucestershire. At all events, we venture to hope that the dements first turned out will be those able to pay the higher rates, and who have no difficulty in finding accommodation in well-managed private asylums; and that the poorer cases who would too surely gravitate to the county asylums will be retained. The committee say that the medical superintendent has conducted the establishment to their entire satisfaction.

---

### THE ROYAL ASYLUM, MONTROSE.

There was an increase of six patients during the year, the number at at the close of their financial year being 561. The admissions were 118, the recoveries 45, and the deaths 47. The recoveries stand at 38·13, and the deaths at 8·51; both of these being close of the general average. Dr. Howden suggests the geographical distribution of insanity as an interesting subject for inquiry, and he gives us some facts, difficult to explain, as to the prevalence of general paralysis in the districts from which his asylum draws its patients. In the year 1860 there was not a single instance of this disease among the 300 patients then resident at Montrose. Since the year 1860 the percentage of general paralytics among the admissions has been: From Arbroath, 2 per cent; from Brechin, 0·66 per cent.; from Forfar, 0·5 per cent.; from Montrose, 4·36 per cent.; Forfarshire, 0·88 per cent.; Kincardine, 0.94; Caithness, 1·2 per cent.; Shetland, 1 per cent.; and Orkney none. Dr. Howden does not attempt to explain the pre-eminence of Montrose in general paralysis, nor why the district was free from it in 1860 and possesses it now. It was once suggested to us that general paralysis was a parasitic disease, and certainly the above facts lend some countenance to the idea. It is curious, too, that it is almost unknown in Ireland. Lectures were delivered by Dr. Havelock to the staff of nurses and attendants, and nine of them passed the examinations of the Medico Psychological Association, and obtained the certificates of efficiency in nursing. Patients in humble circumstances belonging to the counties of Forfar and Kincardine are received at £25 per annum. The intermediate rate is £42, and the higher rates are from £63 to £210. There is a detached villa standing in its own grounds open to ladies, who pay £105. The Commissioners say "the inmates are not subject to any irksome discipline, and many of them have a large amount of liberty. Forty-eight are on parole beyond the grounds, and seventy-six on parole within the grounds." The committee "express their continued confidence in Dr. Howden and his assistants." We should like to have had an opportunity of examining the financial tables.

